# Leisure-time physical activity across adulthood and biomarkers of cardiovascular disease at age 60–64: A prospective cohort study

**DOI:** 10.1016/j.atherosclerosis.2017.11.019

**Published:** 2018-02

**Authors:** Ahmed Elhakeem, Emily T. Murray, Rachel Cooper, Diana Kuh, Peter Whincup, Rebecca Hardy

**Affiliations:** a MRC Unit for Lifelong Health and Ageing at UCL, London, UK; b Musculoskeletal Research Unit, Translational Health Sciences, Bristol Medical School, University of Bristol, Bristol, UK; c Department of Epidemiology and Public Health, University College London, London, UK; d Population Health Research Institute, St George's University of London, London, UK

**Keywords:** Atherosclerosis, Biomarkers, Cardiovascular disease, Exercise, Physical activity

## Abstract

**Background and aims:**

This study examined associations between leisure-time physical activity (LTPA) across adulthood (from age 36) and cardiovascular disease (CVD) biomarkers at age 60–64.

**Methods:**

LTPA was reported by study participants from the MRC National Survey of Health and Development at ages 36, 43, 53 and 60–64 (n = 1754) and categorised as inactive, moderately active (1–4/month) or most active (5+/month) at each age. Linear regression was used to examine associations between a cumulative adulthood LTPA score (range = 0–8), and change in LTPA between ages 36 and 60–64 (i.e. always inactive, became inactive, became active, always active) and inflammatory [C-reactive protein (CRP), interleukin-6 (IL-6)], endothelial [tissue-Plasminogen Activator (t-PA), E-selectin] and adipokine [leptin, adiponectin] measures extracted from overnight fasting blood samples at age 60–64.

**Results:**

The more active a participant was over adulthood, the better their biomarker profile, e.g. fully-adjusted difference in t-PA (both sexes) and adiponectin (women) per unit increase in the LTPA score (95% confidence interval) = −2.2% (−3.6; −0.8) and 2.0% (0.2; 3.8). Those that became active at age 60–64 showed slightly healthier biomarker profiles than those that became inactive [e.g. fully-adjusted difference in IL-6 = −9.9% (−23.9; 4.1) *vs.* −3.8% (−12.4; 4.8)], although the best profiles were seen for those always active [IL-6: −15.0% (−24.2; −5.7)], when compared with the always inactive group.

**Conclusions:**

Greater accumulation of LTPA across adulthood was associated with a more favourable CVD biomarker profile in early old age. Earlier uptake and long-term maintenance of LTPA may provide the greatest benefits for CVD prevention.

## Highlights

• Greater LTPA over 28 years related to healthier atherosclerotic biomarker profiles.
• Taking up LTPA related to better biomarker levels than staying inactive over life.
• Differences in biomarker levels by LTPA were only partly mediated by body size.
• Earlier uptake and long-term maintenance of LTPA may provide greatest benefits.

## 1 Introduction

Leisure-time physical activity (LTPA) of moderate-to-vigorous intensity has consistently been shown to lower risk of cardiovascular disease (CVD) [1], [2] and premature mortality from CVD [2], [3], [4]; the world's leading cause of death [5], [6], [7]. The mechanisms underlying the relationships between LTPA and CVD outcomes are not fully understood, but are thought to be partly due to beneficial effects of physical activity on endothelial function and emerging biomarkers of atherosclerosis, such as inflammatory markers and adipokines [8], [9], [10], [11], [12], [13], [14], [15].

Evidence of how physical activity impacts atherosclerotic biomarkers is primarily based on intervention studies. Most widely investigated are the anti-inflammatory effects of physical activity with consistent findings of decreases in C-reactive protein (CRP) and interleukin-6 (IL-6) following brief exercise interventions [11], [12]. Intervention studies also suggest that exercise-induced fat loss can lower leptin and increase adiponectin levels [8]. Physical activity has also been reported to improve the endothelium's ability to release tissue-plasminogen activator (t-PA) but its effects on E-selectin are unclear [9].

These short-term intervention studies are unable to provide evidence on whether benefits of LTPA remain in the longer term and whether changes in LTPA across life are associated with these biomarkers. Only a few observational studies have examined associations between patterns of physical activity across adulthood and CVD biomarkers. For example, 60-79 year-old men from the British Regional Heart study (BRHS), who increased LTPA from baseline levels reported four [16] and twenty [17] years earlier, had lower CRP and t-PA than those least active at both assessments, and similar t-PA but higher CRP when compared with men who were most active at both time points [16], [17]. Likewise, findings from the Whitehall II Study showed that, over a 10-year period, both consistent, and improvements in, adherence to recommended LTPA (2.5 h/week of moderate-to-vigorous physical activity) were associated with lower levels of pro-inflammatory markers (CRP and IL-6) [18].

However, studies with longer follow-up and more repeated assessments of LTPA over follow-up and, those which consider inflammatory as well as other biomarkers of CVD are needed to investigate how benefits of LTPA might accumulate across adulthood, and how changes in LTPA might influence CVD biomarkers. The aim of this study was therefore to investigate associations between LTPA assessed four times over 28 years in adulthood with inflammatory, endothelial and adipokine markers of CVD measured in early old age. We hypothesised that greater LTPA at each age, and greater accumulation of LTPA would be associated with more favourable CVD biomarker concentrations at age 60–64, and that those who become active would have more favourable levels than those who remained inactive.

## 2 Materials and methods

### 2.1 Study design

The MRC National Survey of Health and Development (NSHD) is a British cohort of 5362 men and women followed up since birth in March 1946 [19], [20]. Between 2006 and 2010 (at age 60–64), a total of 2856 eligible study members (those still alive and with a known address in England, Scotland or Wales) were invited to a clinical research facility (CRF) assessment, or to be visited by a research nurse at home. Invitations were not sent to those who had died (n = 778), emigrated (n = 570), previously withdrawn from the study (n = 594) or were lost to follow-up (n = 564). Of those invited, 2229 (78%) were assessed: 1690 attended a CRF and 539 were seen at home [21].

Ethical approval was obtained from the Greater Manchester Local Research Ethics Committee and the Scotland A Research Ethics Committee. Written, informed consent was obtained from study members for each component of the data collection.

### 2.2 Measurement of adipokines, endothelial and inflammatory markers at age 60-64

Overnight fasting blood samples were taken during the clinical assessment and initially processed at the CRF laboratories. Aliquots were frozen and stored prior to being transferred to the MRC Human Nutrition Research laboratory in Cambridge where analyses of inflammatory marker CRP was processed according to standardised protocols. Analyses of adipokines (leptin and adiponectin), endothelial markers (E-selectin and t-PA) and inflammatory marker IL-6 were undertaken by British Heart Foundation Research Centre in Glasgow. The method and commercial assay plus interassay coefficients of variation are given in Supplementary Data 1.

### 2.3 Adulthood leisure time physical activity (LTPA)

LTPA was reported at ages 36, 43, 53 and 60–64. At age 36, participants were asked how often in the previous month they had participated in 27 different leisure-time activities using a modified Minnesota LTPA questionnaire [22], [23]. At age 43, information was collected on participation in sports, exercise or other vigorous leisure activities in the previous year including for how many months and how often in those months activities were performed [22]. At ages 53 and 60–64, participants reported how often in the previous 4 weeks they participated in any sports, vigorous leisure activities or exercises [22]. At each age, participants were classified as inactive if they reported no participation, moderately active if they participated up to four times, or most active if they participated five or more times in LTPA (in the previous month at age 36, per month at age 43 and in the previous 4 weeks at ages 53 and 60–64) [22].

### 2.4 Covariates

The following covariates were identified *a priori* for inclusion in analyses as they are likely to be associated with both LTPA and CVD biomarkers. Body mass index (BMI) (kg/m^2^) was calculated using heights and weights measured by nurses at age 60–64. Smoking history reported up to age 60–64 was categorised as never smoked, ex-smoker or current smoker. The Registrar General's occupational class at age 53 (or earlier if missing) was used as indicator of socioeconomic position (SEP) and categorised as professional and intermediate (I&II); skilled non-manual (IIINM); skilled manual (IIIM); and semi-skilled and unskilled manual (IV&V). At age 60–64, doctor diagnoses of diabetes, stroke, angina and myocardial infarction were reported, and hypertension (blood pressure ≥ 140/90) was assessed by research nurses using an Omron device.

### 2.5 Statistical analysis

All inflammatory (CRP and IL-6) and endothelial (E-selectin and t-PA) markers and adipokines (adiponectin and leptin) were positively skewed and therefore transformed using the natural logarithm. Sex differences in associations between LTPA at each age and each biomarker were investigated by testing interactions between sex and LTPA using likelihood ratio tests, with sex-stratified analyses performed where evidence of interaction was found.

First, linear regression models were used to examine associations between LTPA at each age and each CVD biomarker (at age 60–64). Second, LTPA responses from ages 36, 43, 53 and 60–64 (coded at each age as 0 = inactive, 1 = moderately active, 2 = most active) were summed to derive a cumulative adulthood LTPA score ranging from 0 (inactive at each age) to 8 (most active at each age) to examine its associations with biomarkers. Deviation from linearity was tested by comparing models with the LTPA score entered as a continuous and as a categorical variable. We tested whether the cumulative LTPA score added information about variation in biomarkers over and above LTPA at 60–64 by using likelihood ratio tests to compare a baseline model including only LTPA at age 60–64 to models with the added cumulative LTPA score.

As study members could be allocated the same cumulative LTPA score despite having different adulthood patterns of LTPA (i.e. having become either more active or inactive), thirdly, we examined associations of change in LTPA by classifying participants based on whether they reported any LTPA at ages 36 and 60–64 as always inactive, always active, became inactive or became active. In this model with LTPA at both ages, we used an interaction term (LTPA at 36 by LTPA at 60–64) to test for deviation from an additive effect. We tested whether change in LTPA between 36 and 60–64 improved model fit by using likelihood ratio tests to compare a baseline model of LTPA at age 60–64 to models with added terms for LTPA at age 36 and the interaction term for LTPA at ages 36 and 60–64.

Age and sex-adjusted differences from the two accumulation models, the second and third analyses described in the previous paragraph, were adjusted in steps for covariates (i.e. SEP, smoking history, BMI, CVD, stroke and diabetes and hypertension). Results were presented as percentage differences since outcomes are logged [24]. Analyses were carried out in STATA 14 (StataCorp, Texas).

### 2.6 Additional and sensitivity analyses

To identify which covariates caused the greatest attenuations in estimates, models were refitted with adjustment in turn for each covariate. To examine mediation by adiposity, we refitted final models before and after adjustment for BMI. We explored residual confounding by earlier BMI by refitting final models with adjustment for BMI at ages 53 and 60–64. To assess the extent of reverse causality whereby those with existing CVD and health problems participate less in LTPA, we repeated analyses after excluding prevalent CVD and diabetes, and refitted final models before and after adjustment for long-term illness, health problems or disability that limits activity (reported at age 60–64). To examine potential effects of diet over and above those of SEP and BMI, final models were refitted before and after adjustment for a marker of healthy diet, the Eating Choices Index, derived from five-day food frequency diaries from a subsample of participants at age 60–64 [25]. Additionally, CRP analyses were repeated after excluding levels >10 mg/l (as these could indicate an acute infection), and further models were also fitted with adjustment for medication use (β-blockers and lipid lowering drugs) at age 60–64. Finally, to investigate bias due to missing data, models were refitted using multiple imputation to account for missing covariates and LTPA. In each case, 20 imputed data sets were obtained via chained equations and estimates were combined using Rubin's rules [26].

## 3 Results

### 3.1 Descriptive statistics

Of 2229 participants with a clinic or home visit at 60–64 years, 2078 (93.2%) had data on at least one CVD biomarker. A further two participants were excluded for not having any LTPA measures at any adult age and 322 for incomplete data on covariates, leaving a total of 1754 participants (51.1% female) with data on least one measure of LTPA and one or more biomarker in addition to complete data on all covariates (Table 1). When compared with those excluded due to missing data on biomarkers, higher proportions of those with data on one or more biomarker were female (51.1% *vs.* 45.7%) and in occupational classes I&II (49.4% *vs.* 33.5%) while lower proportions were current smokers (10.8% *vs.* 14.9%) and had diabetes (6.8% *vs.* 9.7%). Those with data on one or more biomarkers were more active in LTPA across adulthood (e.g. mean adulthood LTPA scores = 3.4 *vs.* 3.0) but there were no differences by BMI, hypertension, or CVD diagnoses.

**Table 1:** Characteristics of study participants from the MRC National Survey of Health and Development with at least one leisure-time physical activity (LTPA) and biomarker measure and data on covariates.

	Overall (n = 1754)	Men (n = 858)	Women (n = 896)	*p* from sex-difference test
**Mean biomarker levels**				
Inflammatory markers				
C-reactive protein (mg/l)	3.9	3.8 (0.3)	3.9 (0.3)	0.7
Interleukin-6 (pg/ml)	2.8	2.9 (0.1)	2.6 (0.09)	0.08
Endothelial markers				
Tissue-plasminogen activator (ng/ml)	10.0	10.7 (0.2)	9.4 (0.2)	<0.001
E-selectin (ng/ml)	39.4	41.4 (0.7)	37.5 (0.6)	<0.001
Adipokines				
Leptin (ng/ml)	18.9	10.1 (0.3)	27.1 (0.9)	<0.001
Adiponectin (μg/ml)	15.0	10.7 (0.3)	19.1 (0.4)	<0.001
**LTPA at each age in adulthood**				
Age 36				0.002
Inactive	538 (33.3)	42.4	57.6	
Moderately active	456 (28.2)	51.8	48.3	
Most active	624 (38.6)	51.7	48.2	
Age 43				0.001
Inactive	812 (48.4)	45.0	55.1	
Moderately active	424 (25.3)	47.6	52.4	
Most active	441 (26.3)	56.2	43.8	
Age 53				0.3
Inactive	746 (44.3)	46.4	53.6	
Moderately active	333 (19.8)	51.1	49.0	
Most active	606 (36.0)	49.0	51.0	
Age 60-64				0.4
Inactive	1088 (62.8)	50.0	50.0	
Moderately active	245 (14.1)	46.1	53.9	
Most active	399 (23.0)	47.4	52.6	
**Mean adulthood LTPA score**	3.33 (0.06)	3.45 (0.09)	3.21 (0.08)	0.04
**LTPA change between 36 and 60**–**64**				<0.001
Inactive at both ages	413 (25.8)	45.5	54.5	
Became inactive at 60-64	596 (37.3)	53.4	46.6	
Became active at 60-64	120 (7.5)	31.7	68.3	
Active at both ages	470 (29.4)	49.8	50.2	

Mean (SE) for continuous data and % for categorical data. LTPA classified from how often in the previous month or 4 weeks they had participated in any sports, vigorous leisure activities or exercises: inactive (0 times), moderately active (1–4 times), most active (≥5 times).

Women had higher levels of adipokines (adiponectin and leptin) than men, while men had higher levels of endothelial markers E-selectin and t-PA (Table 1). A greater proportion of participants were moderately or most active in LTPA at age 36, with higher proportions reporting no LTPA at older ages. More women were inactive at ages 36 and 43 but higher proportions of men became inactive over subsequent follow-up assessments; resulting in approximately two-thirds of men and women categorised as inactive at age 60–64 (Table 1).

### 3.2 LTPA from age 36 and inflammatory markers (CRP and IL-6) at age 60-64

**Fig. 1:**
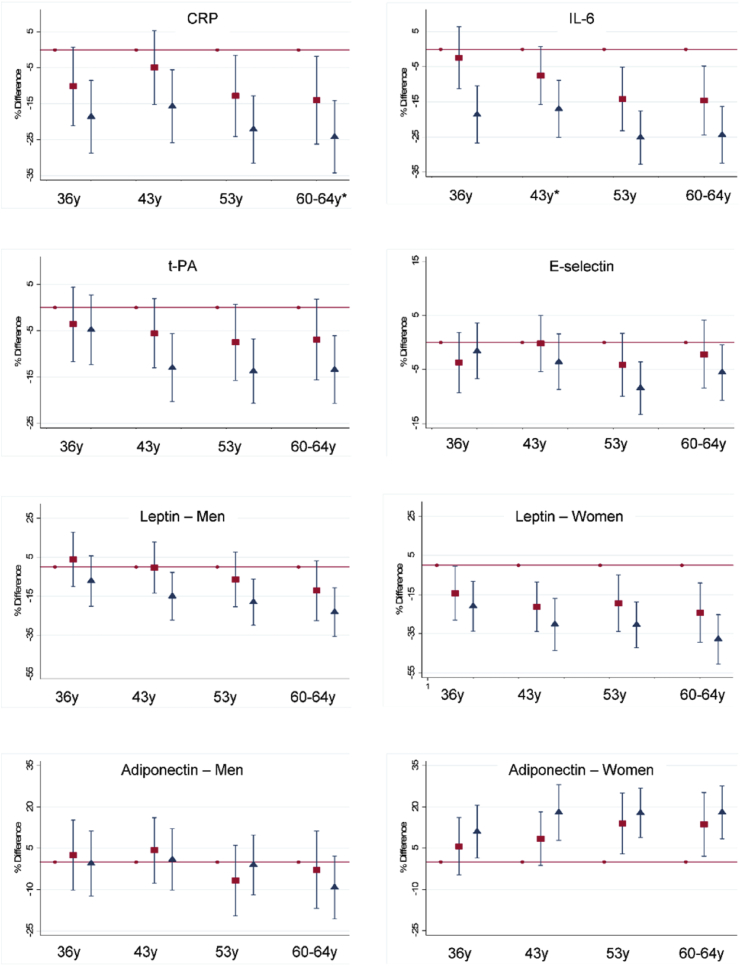
Mean percentage difference in biomarkers at age 60–64 by leisure time physical activity (LTPA) at each age in adulthood. Inflammatory and endothelial markers adjusted for age and sex and adipokines adjusted for age. Reference: no LTPA. Square: moderate LTPA (1–4 times per month). Triangle: most LTPA (5 + times per month). *Sex interaction (*p* ≤ 0.05).

At all ages, more frequent LTPA was related to lower CRP and IL-6 at age 60–64 when compared with no LTPA; there was a trend across groups such that the most active also had lower levels than the moderately active (Fig. 1). For CRP, LTPA at 36 and the interaction between LTPA at 36 and 60–64, and cumulative LTPA score, led to an improved model fit when compared with a model which only included current LTPA (Supplementary Data 3). For IL-6, improvement in model fit was greater for the cumulative LTPA score than the change. Accumulation of LTPA at multiple ages (from age 36 to 60–64) was related to lower CRP and IL-6, and associations were attenuated but not entirely explained by adjustment for covariates (Table 2). Evidence of deviation from linearity was found when examining the association between this cumulative LTPA score and IL-6 (*p* = 0.02); additional investigations revealed that, when compared with an LTPA score of 0–2 (least active across adulthood), those with scores of 5–6 and 7–8 but not 3–4 had lower IL-6. Similarly, when compared with those always inactive at ages 36 and 60–64, those active in LTPA at both ages had lower CRP and IL-6 even after adjustment for covariates (Table 4). Participants that ‘became active’ or ‘became inactive’ had intermediate levels between the ‘always active’ and ‘always inactive’ groups, with those becoming active having slightly better levels than those becoming inactive (Table 4).

**Table 2:** Mean percentage difference (95% confidence intervals) in inflammatory and endothelial markers at age 60–64 by accumulation of leisure-time physical activity (LTPA) across adulthood.

	Inflammatory markers				Endothelial markers			
	C-reactive protein (mg/l) (n = 1510)	*p*	Interleukin-6 (pg/ml) (n = 1506)	*p*	Tissue plasminogen activator (ng/ml) (n = 1324)	*p*	E-selectin (ng/ml) (n = 1506)	*p*
Adulthood LTPA score (0–8): per 1-unit increase								
Model 1	−4.6 (−6.4, −2.7)	<0.001	−5.0 (−6.5, −3.5)	<0.001	−2.9 (−4.3, −1.6)	<0.001	−1.0 (−2.0, −0.1)	0.03
Model 2	−2.4 (−4.3, −0.6)	0.01	−3.5 (−5.0, −2.0)	<0.001	−2.3 (−3.7, −0.9)	0.002	−0.4 (−1.4, 0.5)	0.4
Model 3	−2.4 (−4.3, −0.6)	0.01	−3.4 (−4.9, −1.9)	<0.001	−2.2 (−3.6, −0.8)	0.002	−0.4 (−1.3, 0.6)	0.4

Model 1: adjusted for age and sex. Model 2 also adjusted for body mass index, smoking history and socioeconomic position. Model 3: as for model 2 plus adjustment for hypertension, diabetes, stroke, angina, myocardial infarction. Evidence of deviation from linearity was found between the adult LTPA score and interleukin-6 (*p* = 0.02).

### 3.3 LTPA from age 36 and endothelial markers (t-PA and E-selectin) at age 60-64

More frequent LTPA at each age was related to lower t-PA at age 60–64 when compared with reporting no LTPA, whereas greater LTPA at ages 53 and 60–64 was related to lower E-selectin (Fig. 1). Like the inflammatory markers, groups that were most active had lower levels than the moderately active (Fig. 1). For t-PA, but not E-selectin, the cumulative LTPA score led to an improved model fit when compared with a model that only included concurrent LTPA (Supplementary Data 3). Change in LTPA did not add information for either biomarker (Supplementary Data 3). Cumulative exposure to higher LTPA across adulthood (from age 36 to 60–64) was related to lower t-PA; with associations not entirely explained by adjustment for covariates (Table 2). Accumulation of LTPA across adulthood was weakly associated with lower E-selectin, with this association attenuated after adjustment (Table 2). When examining change in LTPA between ages 36 and 60–64, those active at both ages had lower t-PA levels, compared with those always inactive; even after adjustment for covariates (Table 4). The ‘became active’ had intermediate t-PA levels between the ‘always active’ and ‘always inactive’ groups while the ‘became inactive’ group had t-PA levels like the ‘always inactive’ group. Change in LTPA between 36 and 60–64 was not associated with E-selectin (Table 4).

### 3.4 LTPA from age 36 and adipokines (leptin and adiponectin) at age 60-64

Associations between LTPA and adipokines differed by sex (Supplementary Data 2) so analyses for these outcomes were presented separately for men and women. More frequent LTPA at all ages was associated with lower leptin and higher adiponectin in women whereas for men, only LTPA at ages 53 and 60–64 was associated with leptin, and no associations were found with adiponectin (Fig. 1). The cumulative LTPA score (but not LTPA change from 36) led to an improved model fit over and above current LTPA for both leptin and adiponectin (Supplementary Data 3). Using accumulation models, greater LTPA across adulthood was associated with lower leptin in men and, more strongly, in women, and with higher adiponectin in women only (Table 3). These associations were slightly attenuated, but not fully explained, after adjustment. Evidence of deviation from linearity was found between the adult LTPA score and leptin in men (*p* = 0.009). Additional investigations revealed that, when compared with an LTPA score of 0–2 (least active across adulthood), those with scores of 5–6 and 7–8 but not 3–4 had lower leptin levels.

**Table 3:** Mean percentage difference (95% confidence intervals) in adipokines at age 60–64 by accumulation of leisure-time physical activity (LTPA) across adulthood.

	Leptin (ng/ml)				Adiponectin (ug/ml)			
	Men (n = 724)	*p*	Women (n = 783)	*p* -value	Men (n = 723)	*p*	Women (n = 783)	*p* -value
Adulthood LTPA score (0–8): per 1-unit increase								
Model 1	−3.9 (−6.3, −1.6)	0.001	−7.8 (−10.1, −5.5)	<0.001	−0.6 (−2.7, 1.5)	0.5	4.2 (2.4, 6.0)	<0.001
Model 2	−4.4 (−6.2, −2.6)	<0.001	−2.4 (−4.2, −0.6)	0.01	−0.8 (−3.0, 1.3)	0.4	2.1 (0.3, 3.9)	0.02
Model 3	−4.2 (−6.0, −2.4)	<0.001	−2.4 (−4.2, −0.5)	0.01	−1.1 (−3.2, 1.1)	0.3	2.0 (0.2, 3.8)	0.03

Model 1: adjusted for age. Model 2 also adjusted for body mass index, smoking history and socioeconomic position. Model 3: as for model 2 plus adjustment for hypertension, diabetes, stroke, angina, myocardial infarction. Evidence of deviation from linearity was found between the adult LTPA score and leptin in men (*p* = 0.009).

When compared with ‘always inactive’ at 36 and 60–64, those who participated in LTPA at both ages had lower leptin levels even after adjustment for covariates (Table 4). Groups that ‘became active’ or ‘became inactive’ had intermediate levels between the ‘always active’ and ‘always inactive’ groups (Table 4). Only men who were active at both ages had lower leptin levels than those inactive at both ages (*p* = 0.09 for LTPA at age 36 by LTPA at age 60–64 interaction term). Women who remained active and, to a lesser extent, women who became active had higher adiponectin than those ‘always inactive’ but differences were reduced by adjustment (Table 4).

**Table 4:** Mean percentage difference (95% confidence intervals) in biomarkers at age 60–64 by change in leisure-time physical activity (LTPA) between ages 36 and 60–64.

	Always inactive	Became inactive	Became active	Always active	*p* (overall association)
C-reactive protein (n = 1592)					
Model 1	0.0	−13.2 (−24.2, −2.2)	−20.6 (−38.4, −2.8)	−27.9 (−39.5, −16.3)	<0.001
Model 2	0.0	−7.5 (−18.1, 3.0)	−8.5 (−25.7, 8.7)	−15.6 (−27.0, −4.2)	0.06
Model 3	0.0	−7.7 (−18.3, 2.9)	−8.5 (−25.7, 8.7)	−15.6 (−27.0, −4.1)	0.07

Interleukin-6 (n = 1585)					
Model 1	0.0	−8.6 (−17.4, 0.3)	−20.3 (−34.6, −5.9)	−25.2 (−34.5, −15.9)	<0.001
Model 2	0.0	−4.0 (−12.6, 4.6)	−10.3 (−24.3, 3.7)	−15.7 (−24.9, −6.4)	0.005
Model 3	0.0	−3.8 (−12.4, 4.8)	−9.9 (−23.9, 4.1)	−15.0 (−24.2, −5.7)	0.009

Tissue plasminogen activator (n = 1388)					
Model 1	0.0	0.9 (−7.3, 9.1)	−5.0 (−18.1, 8.0)	−12.5 (−21.0, −4.0)	0.004
Model 2	0.0	2.8 (−5.2, 10.9)	−0.8 (−13.8, 12.1)	−7.8 (−16.3, 0.8)	0.05
Model 3	0.0	2.7 (−5.4, 10.8)	−1.2 (−14.2, 11.7)	−7.8 (−16.3, 0.8)	0.06

E-selectin (n = 1585)					
Model 1	0.0	−1.9 (−7.6, 3.7)	−5.0 (−14.1, 4.2)	−5.8 (−11.7, 0.2)	0.2
Model 2	0.0	0.0 (−5.6, 5.5)	−1.3 (−10.3, 7.7)	−1.7 (−7.7, 4.2)	0.9
Model 3	0.0	0.1 (−5.5, 5.6)	−1.5 (−10.5, 7.5)	−1.5 (−7.5, 4.4)	0.9

Leptin – men (n = 771)					
Model 1	0.0	8.3 (−5.4, 22.0)	1.3 (−25.4, 28.0)	−16.1 (−30.6, −1.5)	0.003
Model 2	0.0	−0.4 (−10.8, 10.0)	−2.4 (−22.7, 18.0)	−19.5 (−3.7, −8.3)	<0.001
Model 3	0.0	−0.5 (−10.9, 9.9)	1.1 (−21.3, 19.1)	−18.3 (−29.5, −7.2)	0.001

Leptin – women (n = 815)					
Model 1	0.0	−11.5 (−25.3, 2.4)	−28.4 (−48.4, −8.4)	−42.7 (−57.3, −28.2)	<0.001
Model 2	0.0	7.4 (−2.9 17.7)	−1.2 (−16.2, 13.7)	−10.7 (−21.9, 0.4)	0.007
Model 3	0.0	7.3 (−3.0, 17.7)	−1.3 (−16.2, 13.7)	−10.6 (−21.7, 0.6)	0.008

Adiponectin – men (n = 770)					
Model 1	0.0	0.9 (−11.7, 13.4)	−14.5 (−38.9, 9.9)	−6.5 (−19.8, 6.8)	0.4
Model 2	0.0	2.9 (−9.5, 15.4)	−15.1 (−39.3, 9.2)	−7.5 (−20.9, 5.9)	0.2
Model 3	0.0	1.6 (−10.6, 14.0)	−17.2 (−41.1, 6.8)	−10.2 (−23.5, 3.0)	0.1

Adiponectin – women (n = 815)					
Model 1	0.0	7.5 (−3.1, 18.0)	18.3 (3.1, 33.6)	21.5 (10.4, 32.5)	<0.001
Model 2	0.0	0.1 (−10.1, 10.4)	7.5 (−7.4, 22.3)	8.9 (−2.2, 19.9)	0.3
Model 3	0.0	−0.6 (−10.9, 9.6)	7.9 (−7.0, 22.7)	8.2 (−2.8, 19.3)	0.3

Model 1: adjusted for age (and sex). Model 2 also adjusted for body mass index, smoking history and socioeconomic position. Model 3: as for model 2 plus adjustment for hypertension, diabetes, stroke, angina, myocardial infarction. There was no evidence of age 36 by age 60–64 LTPA interactions, except for a suggestion of an interaction for leptin in men (*p* = 0.09).

### 3.5 Additional and sensitivity analyses

The greatest reductions in biomarker differences were seen after adjustment for BMI, smoking history and SEP (model 2 in Table 2, Table 3, Table 4). Further analyses showed that BMI caused the greatest attenuation in estimates, suggesting associations were predominantly mediated by adiposity. Adjustment for BMI recorded at ages 53 and 60–64 had no influence on findings. Results were similar after adjustment for long-term limiting illness (Supplementary Data 4) and after exclusion of participants with CVD and diabetes (n = 233). Additional adjustment for diet had little influence on associations (Supplementary Data 5). Excluding CRP levels >10 mg/l (n = 230), and further adjustment for medication use (up to 200 participants were taking β-blockers and 400 taking lipid lowering drugs) also did not influence findings. Lastly, similar results were found after multiple imputation (Supplementary Data 6).

## 4 Discussion

### 4.1 Main findings

Findings from this British cohort study showed that greater participation in LTPA over 28 years across adulthood was associated with more favourable levels of CVD biomarkers in early old age, and highlight the added value of prior LTPA for explaining variations in biomarkers over current LTPA alone. More frequent LTPA at ages 36, 43, 53 and 60–64, and therefore greater accumulation of LTPA across adulthood, was associated with lower CRP, IL-6, t-PA and leptin levels in both men and women, and higher adiponectin levels in women only. Consistent with a model of accumulation, the ‘always active’ group showed the best biomarker levels, compared to the groups with less consistent LTPA across adulthood. The group who became active showed better biomarker profiles than the group who became inactive. Associations were only partly attenuated following adjustment for a range of covariates, with adjustment for BMI causing the greatest reduction in differences.

### 4.2 Relation to other studies

Our findings are consistent with the few other epidemiological studies that have examined associations between patterns of LTPA and CVD biomarkers, but over a shorter time period or later in life. These include findings that older men from BRHS that maintained participation in LTPA over up to 20-years follow-up had the lowest levels of CRP and t-PA, with those least active at both assessment having the highest levels [16], [17]. Our results are also similar to findings from the Whitehall II Study, which showed that participants who were regularly active at all 3 assessments over the 10-year period had the lowest levels of the pro-inflammatory markers CRP and IL-6 [18]. Likewise, and consistent with our findings, CVD outpatients from the Heart and Soul Study, that were active in LTPA at 5-year follow-up, having also been previously active at baseline, had lowest levels of CRP and IL-6 [27]. Our study is important because it shows that LTPA patterns as early as mid-adulthood were associated with a wide range of CVD biomarkers 28 years later.

### 4.3 Explanation of findings

These findings suggest that long-term LTPA participation may influence physiological and biochemical functions of blood vessels and lend support to an accumulation of additive effects hypothesis whereby continued exposure to greater LTPA cumulatively benefits subsequent levels of CVD biomarkers. The endothelium is directly (and indirectly through effects on other body tissues) involved in maintaining vascular homeostasis and many of its functions are responsive to physical activity [9], [12], [13], [14], [28].

CRP and IL-6 are the most well-known pro-inflammatory molecules and at higher concentrations are considered strong predictors of CVD [29]. Acute exposure to physical activity is known to increase anti-inflammatory and reduce pro-inflammatory cytokines which play a main role in arterial stiffening [11], [12], [14] and our findings suggest that minimising the amount of inflammatory response is also one benefit of long-term LTPA. Likewise, t-PA, an endothelial-specific glycoprotein critical to fibrinolysis which at high circulating levels is predictive of CVD [30], appears responsive to long-term LTPA.

Our findings also suggest a beneficial effect of long-term LTPA on leptin in men and women and adiponectin in women only. Leptin and adiponectin are adipose tissue-based cytokines whose levels are altered in endothelial dysfunction and which are thought to be involved in CVD aetiology [31]; increases in adiponectin are protective and in leptin are damaging to vascular homeostasis [31]. Intervention studies have reported inconsistent relationships between exercise and adiponectin [8] and our finding that adulthood LTPA was related to adiponectin in women only requires investigation in other long-term studies. Likewise, although a weak inverse association between greater accumulation of LTPA across adulthood and lower E-selectin was observed, there was little evidence overall of association between LTPA and E-selectin which is similar to a review of intervention studies [9].

In the present analyses, adjustment for current BMI resulted in the greatest attenuation of estimates although it did not fully explain associations. This suggests that BMI may be one route, but not the only route, through which LTPA benefits cardiovascular health [32] and is consistent with studies showing persisting associations after adjustment for body size [17], [18]. Evidence from longitudinal studies suggests that moderate-to-vigorous physical activity, which makes up the majority of time spent in LTPA [33], can help prevent excess body fat [34], [35]. Furthermore, the cardiovascular benefits of LTPA have been shown at all body weights including overweight and obesity [36] which are established CVD risk factors [37]. In addition to our current findings, we recently showed that longer time spent overweight or obese across life in NSHD was associated with more adverse levels of inflammatory markers and adipokines at age 60–64 [38] which, in light of our current findings, suggest that the benefits of LTPA and BMI on these biomarkers likely involves one pathway from LTPA through BMI as well as additional pathways from BMI and from LTPA.

Other effects of physical activity on endothelial function which have been described include enhanced nitric oxide production (responsible for vasodilation, lowering peripheral resistance and increasing perfusion) and reduced concentrations of vasoconstrictor agents [9], [13], [14]. It is also possible that LTPA improves mobilisation of endothelial progenitor cells and mesenchymal stem cells from bone marrow, both which have the potential for vascular regeneration and endothelial repair [28]. These findings along with the associations reported here may explain how long-term participation in LTPA contributes to reducing age-related structural changes seen in atherosclerotic arteries and subsequent CVD risk [39], [40], [41]. For example, taking up or maintaining LTPA over 8 years was found to be associated with lower arterial stiffness in middle-aged Swiss adults [39].

### 4.4 Strengths and limitations

This study has several important strengths. These include the longitudinal design which results in less susceptibility to reverse causation and the repeated reporting of LTPA over almost three decades of adulthood to examine how long-term patterns of LTPA relate to a wide range of biomarkers of CVD risk. Adjustment for important and prospectively ascertained covariates, including BMI and SEP, is another notable strength. However, some limitations should also be noted. There were a limited number of participants (n = 120) who became active at age 60–64 having been inactive at age 36 which may have reduced statistical power to detect differences by change in LTPA status. Additionally, the categorisation of LTPA was relatively crude in that it did not differentiate between higher levels of LTPA (above participating five or more times/month). LTPA was self-reported and can be subject to recall and misclassification errors but these type of data are suitable for providing information on certain activity domains like LTPA [42]. Further, the LTPA measures used here were found to rank NSHD participants by levels of physical activity similarly to data from activity monitors [43], [44]. Moreover, we only examined LTPA however, other activity domains such as active transport may be beneficially related to cardiovascular biomarkers [45] and may form important components of overall PA.

Residual confounding by earlier BMI could explain why adjustment for current BMI did not fully explain associations; however current BMI was previously shown to be an adequate proxy for lifetime BMI in this cohort [46], and adjustment for BMI recorded at ages 53 and 60–64 had no influence on findings. Further, residual confounding due to other unmeasured factors such as urbanicity/rurality is possible. Finally, doctor diagnoses were self-reported and could be prone to errors however, these measures have been validated against medical records in NSHD and other similar aged cohorts [47], [48].

### 4.5 Conclusions

This long-term prospective follow-up study of British men and women found that greater participation in LTPA and maintaining participation across adulthood were associated with a more favourable CVD biomarker profile in early old age. These findings support an important role for continued long-term participation in LTPA in the prevention of CVD. Of further importance to public health interventions is that our findings suggest that taking up LTPA in early old age may also have benefits for cardiovascular health.

## Conflict of interest

The authors declared they do not have anything to disclose regarding conflict of interest with respect to this manuscript.

## Financial support

This work was supported by the UK Medical Research Council [MC_UU_12019/1 and G1001143]. The funder had no role in the writing of the manuscript and played no part in the decision to submit it for publication.

## Author contributions

Study design: AE, ETM, RC, DK, PHW, RH. Data analysis: AE, ETM. Data interpretation: AE, ETM, RC, DK, PHW, RH. AE and ETM produced the first manuscript draft and all authors read and approved its final version.
