# Supplementary material for: Leisure-time physical activity across adulthood and biomarkers of cardiovascular disease at age 60–64: A prospective cohort study
Source: Atherosclerosis. 2018 Feb;269:279–87. doi: 10.1016/j.atherosclerosis.2017.11.019 (PMC5825380; doi:10.1016/j.atherosclerosis.2017.11.019)
Supplement: Supplementary material 1 [file mmc1.docx]

**Supplementary table 1** Methods and interassay coefficients (CV) of variation for cardiovascular disease biomarkers assessed from blood samples.

| Cardiovascular disease biomarker | Units | Assay/Method | CV (%) |
| --- | --- | --- | --- |
|  |  |  |  |
| E-selectin | ng/ml | High sensitivity ELISA | <10.0% |
| C-reactive protein (CRP) | mg/l | Particle-enhanced immunoturbidimetric assay | 4.3% at 3.4 mg/L 1.8% at 11.9 mg/L |
| Interleukin-6 (IL6) | pg/ml | Enzyme-linked immunosorbent assay (ELISA) | 6.5% |
| Tissue plasminogen activator (tPA) | ng/ml | ELISA | 6.6% |
| Leptin | ng/ml | In-house radioimmunoassay validated against commercially available assays | <10.0% |
| Adiponectin | *μ*g/ml | ELISA | <7.5% |

* Any results that fell below the lower detection limit of the assay were assigned a notional value, obtained by dividing the lower detection limit by the square root of 2.
