# Supplementary material for: Leisure-time physical activity across adulthood and biomarkers of cardiovascular disease at age 60–64: A prospective cohort study
Source: Atherosclerosis. 2018 Feb;269:279–87. doi: 10.1016/j.atherosclerosis.2017.11.019 (PMC5825380; doi:10.1016/j.atherosclerosis.2017.11.019)
Supplement: Supplementary material 2 [file mmc2.docx]

**Supplementary table 2** P-values for sex interactions with LTPA, fitted separately for each biomarker and age.

|  | **Adipokines** | | **Endothelial markers** | | **Inflammatory markers** | |
| --- | --- | --- | --- | --- | --- | --- |
| **LTPA** | *Adiponectin (ug/ml)* | *Leptin* (ng/ml) | *Eselectin (ng/ml)* | *Tissue plasminogen activator* (ng/ml) | *C-reactive protein (mg/l)* | *Interleukin-6 (pg/ml)* |
| 36 years | **0.01** | **<0.01** | 0.88 | 0.24 | 0.08 | 0.20 |
| 43 years | **0.03** | **0.03** | 0.81 | 0.88 | 0.12 | **0.05** |
| 53 years | **<0.01** | 0.14 | 0.37 | 0.64 | 0.13 | 0.30 |
| 63 years | **<0.01** | **0.02** | 0.95 | 0.76 | **0.03** | 0.81 |
