# Supplementary material for: Leisure-time physical activity across adulthood and biomarkers of cardiovascular disease at age 60–64: A prospective cohort study
Source: Atherosclerosis. 2018 Feb;269:279–87. doi: 10.1016/j.atherosclerosis.2017.11.019 (PMC5825380; doi:10.1016/j.atherosclerosis.2017.11.019)
Supplement: Supplementary material 3 [file mmc3.docx]

|  | *P* from likelihood ratio tests of model with LTPA at age 60-64 versus: | | |
| --- | --- | --- | --- |
|  | model with + age 36 LTPA | model with + age 36-by age 60-64 LTPA interaction | Model with + cumulative adult LTPA score |
| *Inflammatory markers* |  |  |  |
| CRP (mg/l) | 0.002 | 0.007 | <0.001 |
| IL-6 (pg/ml) | 0.09 | 0.2 | <0.001 |
|  |  |  |  |
| *Endothelial markers* |  |  |  |
| t-PA (ng/ml) | 0.8 | 0.4 | 0.05 |
| E-selectin (ng/ml) | 0.9 | 0.9 | 0.2 |
|  |  |  |  |
| *Adipokines* |  |  |  |
| Leptin (ng/ml) | 0.2 | 0.1 | <0.001 |
| Adiponectin (ug/ml) | 0.06 | 0.2 | 0.02 |

**Supplementary table 3** P-values from likelihood ratio tests comparing models with age 60-64 LTPA to models with additional terms for (a) age 36 LTPA, (b) age 36-by-age 60-64 LTPA interaction, and (c) cumulative adulthood LTPA score.

Adjusted for age and sex
