# Supplementary material for: Leisure-time physical activity across adulthood and biomarkers of cardiovascular disease at age 60–64: A prospective cohort study
Source: Atherosclerosis. 2018 Feb;269:279–87. doi: 10.1016/j.atherosclerosis.2017.11.019 (PMC5825380; doi:10.1016/j.atherosclerosis.2017.11.019)
Supplement: Supplementary material 4 [file mmc4.docx]

**Supplementary table 4a** Mean percentage difference in inflammatory and endothelial markers at age 60-64 by accumulation of leisure-time physical activity (LTPA) across adulthood – after adjustment for long-term illness, health problem or disability.

|  | **Inflammatory markers** | | | |  | **Endothelial markers** | | | |  |
| --- | --- | --- | --- | --- | --- | --- | --- | --- | --- | --- |
|  | *C-reactive protein (mg/l) (n=1508)* | *P* | *Interleukin-6 (pg/ml) (n=1504)* | *P* |  | *Tissue plasminogen activator* (ng/ml) (n=1322) | *P* | *E-selectin (ng/ml) (n=1504)* | *P* |  |
| *Adulthood LTPA score (0-8): per 1-unit increase* |  |  |  |  |  |  |  |  |  |  |
| Model A | -2.5 (-4.3, -0.6) | 0.009 | -3.4 (-4.9, -1.9) | <0.001 |  | -2.3 (-3.7, -0.9) | 0.001 | -0.4 (-1.3, 0.6) | 0.5 |  |
| Model B | -2.3 (-4.2, -0.5) | 0.01 | -3.3 (-4.8, -1.8) | <0.001 |  | -2.3 (-3.7, -0.9) | 0.002 | -0.4 (-1.3, 0.6) | 0.4 |  |

Model A: adjusted for age, sex, BMI, smoking history and socioeconomic position, hypertension, diabetes, stroke, angina, myocardial infarction. Model B: as for model 2 plus adjustment for long-term illness, health problem or disability.

**Supplementary table 4b** Mean percentage difference in adipokines at age 60-64 by accumulation of leisure-time physical activity (LTPA) across adulthood – after adjustment for long-term illness, health problem or disability.

|  | **Leptin (ng/ml)** | | | |  | **Adiponectin (ug/ml)** | | | |
| --- | --- | --- | --- | --- | --- | --- | --- | --- | --- |
|  | *Men (n=724)* | *P* | *Women (n=781)* | *p-value* |  | *Men (n=723)* | *P* | *Women (n=781)* | *p-value* |
| *Adulthood LTPA score (0-8): per 1-unit increase* |  |  |  |  |  |  |  |  |  |
| Model A | -4.2 (-6.0, -2.4) | <0.001 | -2.4 (-4.3, -0.6) | 0.009 |  | -1.1 (-3.2, 1.1) | 0.3 | 2.0 (0.2, 3.8) | 0.03 |
| Model B | -4.1 (-6.0, -2.3) | <0.001 | -2.4 (-4.3, -0.6) | 0.009 |  | -1.1 (-3.2, 1.0) | 0.3 | 2.0 (0.2, 3.9) | 0.03 |

Model A: adjusted for age, sex, BMI, smoking history and socioeconomic position, hypertension, diabetes, stroke, angina, myocardial infarction. Model B: as for model 2 plus adjustment for long-term illness, health problem or disability.

**Supplementary table 4c** Mean percentage difference in biomarkers at age 60-64 by change in leisure-time physical activity (LTPA) between ages 36 and 60-64 – after adjustment for long-term illness, health problem or disability.

|  | Always inactive | Became inactive | Became active | Always active | p (overall association) |
| --- | --- | --- | --- | --- | --- |
| *C-reactive protein (n=1589)* |  |  |  |  |  |
| Model A | 0.0 | -7.8 (-18.4, 2.8) | -7.8 (-25.1, 9.4) | -15.6 (-27.1, -4.2) | 0.06 |
| Model B | 0.0 | -7.5 (-18.0, 3.0) | -6.6 (-23.8, 10.6) | -14.4 (-25.7, -3.0) | 0.1 |
|  |  |  |  |  |  |
| *Interleukin-6 (n=1582)* |  |  |  |  |  |
| Model A | 0.0 | -3.8 (-12.4, 4.8) | -10.0 (-24.0, 4.0) | -15.1 (-24.3, -5.8) | 0.008 |
| Model B | 0.0 | -3.7 (-12.2, 4.9) | -9.2 (-23.2, 4.8) | -14.3 (-23.5, -5.0) | 0.01 |
|  |  |  |  |  |  |
| *Tissue plasminogen activator (n=1386*) |  |  |  |  |  |
| Model A | 0.0 | 2.8 (-5.3, 10.9) | -1.2 (-14.2, 11.8) | -7.8 (-16.4, 0.8) | 0.05 |
| Model B | 0.0 | 2.8 (-5.3, 10.9) | -1.2 (-14.2, 11.8) | -7.6 (-16.2, 0.9) | 0.06 |
|  |  |  |  |  |  |
| *E-selectin (n=1582)* |  |  |  |  |  |
| Model A | 0.0 | 0.1 (-5.4, 5.7) | -2.0 (-11.0, 7.0) | -1.5 (-7.4, 4.5) | 0.9 |
| Model B | 0.0 | 0.1 (-5.4, 5.7) | -2.1 (-11.1, 7.0) | 1.5 (-7.1, 4.4) | 0.9 |
|  |  |  |  |  |  |
| Leptin – men (n=770) |  |  |  |  |  |
| Model A | 0.0 | -0.5 (-10.9, 9.9) | -0.5 (-20.7, 19.7) | -18.1 (-29.3, -6.9) | 0.001 |
| Model B | 0.0 | -0.4 (-10.8, 10.0) | -0.3 (-20.5, 20.0) | -18.0 (-29.3, -6.8) | 0.001 |
|  |  |  |  |  |  |
| Leptin – women (n=813) |  |  |  |  |  |
| Model A | 0.0 | 7.9 (-2.5, 18.3) | -2.2 (-17.3, 12.9) | -10.9 (-22.1, 0.4) | 0.005 |
| Model B | 0.0 | 7.8 (-2.5, 18.2) | -2.1 (-17.2, 13.0) | -10.6 (-21.8, 0.7) | 0.006 |
|  |  |  |  |  |  |
| Adiponectin – men (n=769) |  |  |  |  |  |
| Model A | 0.0 | 1.5 (-10.8, 13.8) | -17.5 (-41.4, 6.5) | -10.4 (-23.7, 2.9) | 0.1 |
| Model B | 0.0 | 1.4 (-10.9, 13.7) | -17.8 (-41.8, 6.2) | -10.6 (-23.9, 2.7) | 0.1 |
|  |  |  |  |  |  |
| Adiponectin – women (n=813) |  |  |  |  |  |
| Model A | 0.0 | -0.8 (-11.0, 9.4) | 8.5 (-6.4, 23.4) | 8.5 (-2.6, 19.6) | 0.2 |
| Model B | 0.0 | -0.9 (-11.2, 9.3) | 8.7 (-6.2, 23.6) | 9.0 (-2.1, 20.0) | 0.2 |

Model A: adjusted for age, sex, BMI, smoking history and socioeconomic position, hypertension, diabetes, stroke, angina, myocardial infarction. Model B: as for model 2 plus adjustment for long-term illness, health problem or disability.
