# Supplementary material for: Leisure-time physical activity across adulthood and biomarkers of cardiovascular disease at age 60–64: A prospective cohort study
Source: Atherosclerosis. 2018 Feb;269:279–87. doi: 10.1016/j.atherosclerosis.2017.11.019 (PMC5825380; doi:10.1016/j.atherosclerosis.2017.11.019)
Supplement: Supplementary material 5 [file mmc5.docx]

**Supplementary table 5a** Mean percentage difference in inflammatory and endothelial markers at age 60-64 by accumulation of leisure-time physical activity (LTPA) across adulthood – after adjustment for diet.

|  | **Inflammatory markers** | | | |  | **Endothelial markers** | | | |  |
| --- | --- | --- | --- | --- | --- | --- | --- | --- | --- | --- |
|  | *C-reactive protein (mg/l) (n=1317)* | *P* | *Interleukin-6 (pg/ml) (n=1317)* | *P* |  | *Tissue plasminogen activator* (ng/ml) (n=1168) | *P* | *E-selectin (ng/ml) (n=1317)* | *P* |  |
| *Adulthood LTPA score (0-8): per 1-unit increase* |  |  |  |  |  |  |  |  |  |  |
| Model A | -2.1 (-4.1, -0.2) | 0.03 | -3.3 (-4.9, -1.7) | <0.001 |  | -2.3 (-3.7, -0.9) | 0.002 | -0.3 (-1.3, 0.7) | 0.6 |  |
| Model B | -1.9 (-3.8, 0.1) | 0.06 | -3.0 (-4.7, -1.4) | <0.001 |  | -2.0 (-3.4, -0.5) | 0.007 | -0.1 (-1.1, 0.9) | 0.8 |  |

Model A: adjusted for age, sex, BMI, smoking history and socioeconomic position, hypertension, diabetes, stroke, angina, myocardial infarction. Model B: as for model 2 plus adjustment for diet at age 60-64 (the Eating Choices Index).

**Supplementary table 5b** Mean percentage difference in adipokines at age 60-64 by accumulation of leisure-time physical activity (LTPA) across adulthood – after adjustment for diet.

|  | **Leptin (ng/ml)** | | | |  | **Adiponectin (ug/ml)** | | | |
| --- | --- | --- | --- | --- | --- | --- | --- | --- | --- |
|  | *Men (n=623)* | *P* | *Women (n=695)* | *p-value* |  | *Men (n=623)* | *P* | *Women (n=695)* | *p-value* |
| *Adulthood LTPA score (0-8): per 1-unit increase* |  |  |  |  |  |  |  |  |  |
| Model A | -3.8 (-5.7, -1.8) | <0.001 | -2.2 (-4.1, -0.3) | 0.03 |  | -0.6 (-2.8, 1.6) | 0.6 | 2.9 (1.0, 4.8) | 0.003 |
| Model B | -3.6 (-5.6, -1.6) | <0.001 | -1.7 (-3.7, 0.2) | 0.08 |  | -0.5 (-2.8, 1.7) | 0.7 | 2.6 (0.7, 4.6) | 0.008 |

Model A: adjusted for age, BMI, smoking history and socioeconomic position, hypertension, diabetes, stroke, angina, myocardial infarction. Model B: as for model 2 plus adjustment for diet at age 60-64 (the Eating Choices Index).

**Supplementary table 5c** Mean percentage difference in biomarkers at age 60-64 by change in leisure-time physical activity (LTPA) between ages 36 and 60-64 – after adjustment for diet.

|  | Always inactive | Became inactive | Became active | Always active | p (overall association) |
| --- | --- | --- | --- | --- | --- |
| *C-reactive protein (n=1382)* |  |  |  |  |  |
| Model A | 0.0 | -7.4 (-18.8, 4.0) | -8.9 (-27.0, 9.2) | -14.7 (-27.0, -2.7) | 0.1 |
| Model B | 0.0 | -7.2 (-18.6, -4.2) | -8.0 (-26.1, 0.1) | -13.2 (-25.3, -1.1) | 0.2 |
|  |  |  |  |  |  |
| *Interleukin-6 (n=1379)* |  |  |  |  |  |
| Model A | 0.0 | -4.9 (-14.3, 4.5) | -9.4 (-24.3, 5.5) | -13.2 (-23.1, -3.3) | 0.06 |
| Model B | 0.0 | -4.7 (-14.0, 4.7) | -8.4 (-23.3, 6.4) | -11.6 (-21.6, -1.6) | 0.1 |
|  |  |  |  |  |  |
| *Tissue plasminogen activator (n=1219*) |  |  |  |  |  |
| Model A | 0.0 | 3.5 (-4.9, 11.9) | -1.0 (-14.1, 12.0) | -7.6 (-16.4, 1.1) | 0.04 |
| Model B | 0.0 | 3.9 (-4.4, 12.3) | 0.2 (-12.8, 13.2) | -5.7 (-14.5, 3.0) | 0.1 |
|  |  |  |  |  |  |
| *E-selectin (n=1379)* |  |  |  |  |  |
| Model A | 0.0 | -0.9 (-6.9, 5.0) | -1.3 (-10.7, 8.2) | -1.9 (-8.2, 4.4) | >0.9 |
| Model B | 0.0 | -0.8 (-6.8, 5.2) | -0.6 (-10.1, 8.8) | -0.8 (-7.2, 5.5) | >0.9 |
|  |  |  |  |  |  |
| Leptin – men (n=662) |  |  |  |  |  |
| Model A | 0.0 | -3.5 (-14.9, 8.0) | -1.3 (-22.9, 20.2) | -17.9 (-30.1, -5.7) | 0.01 |
| Model B | 0.0 | -3.7 (-15.1, 7.8) | -2.0 (-23.6, 19.5) | -17.2 (-29.5, -5.0) | 0.02 |
|  |  |  |  |  |  |
| Leptin – women (n=718) |  |  |  |  |  |
| Model A | 0.0 | 8.5 (-2.4, 19.4) | 1.9 (-13.6, 17.5) | -10.0 (-21.6, 1.6) | 0.007 |
| Model B | 0.0 | 9.4 (-1.5, 20.2) | 4.7 (-10.9, 20.2) | -7.3 (-19.0, 4.5) | 0.02 |
|  |  |  |  |  |  |
| Adiponectin – men (n=662) |  |  |  |  |  |
| Model A | 0.0 | 4.6 (-8.7, 17.9) | -18.7 (-4.4, 6.2) | -8.2 (-22.3, 5.9) | 0.08 |
| Model B | 0.0 | 4.5 (-8.8, 17.8) | -19.0 (-4.4, 5.9) | -7.9 (-22.1, 6.3) | 0.09 |
|  |  |  |  |  |  |
| Adiponectin – women (n=718) |  |  |  |  |  |
| Model A | 0.0 | 2.8 (-8.1, 13.6) | 9.5 (-6.0, 25.0) | 13.6 (2.0, 25.2) | 0.09 |
| Model B | 0.0 | 2.3 (-8.5, 13.2) | 8.2 (-7.4, 23.8) | 12.3 (0.6, 24.0) | 0.2 |

Model A: adjusted for age, (sex), BMI, smoking history and socioeconomic position, hypertension, diabetes, stroke, angina, myocardial infarction. Model B: as for model 2 plus adjustment for diet at age 60-64 (the Eating Choices Index).
