# Supplementary material for: Leisure-time physical activity across adulthood and biomarkers of cardiovascular disease at age 60–64: A prospective cohort study
Source: Atherosclerosis. 2018 Feb;269:279–87. doi: 10.1016/j.atherosclerosis.2017.11.019 (PMC5825380; doi:10.1016/j.atherosclerosis.2017.11.019)
Supplement: Supplementary material 6 [file mmc6.docx]

**Supplementary table 6a** Mean percentage difference in inflammatory and endothelial markers at age 60-64 by accumulation of leisure-time physical activity (LTPA) across adulthood – after multiple imputation.

|  | **Inflammatory markers** | | | |  | **Endothelial markers** | | | |  |
| --- | --- | --- | --- | --- | --- | --- | --- | --- | --- | --- |
|  | *C-reactive protein (mg/l) (n=2065)* | *P* | *Interleukin-6 (pg/ml) (n=2052)* | *P* |  | *Tissue plasminogen activator* (ng/ml) (n=1798) | *P* | *E-selectin (ng/ml) (n=2052)* | *P* |  |
| *Adulthood LTPA score (0-8): per 1-unit increase* |  |  |  |  |  |  |  |  |  |  |
| Model 1 | -5.2 (-7.0, -3.5) | <0.001 | -3.6 (-5.0, -2.2) | <0.001 |  | -2.4 (-3.7, -1.1) | <0.001 | -1.1 (-2.0, -0.2) | 0.01 |  |
| Model 2 | -2.8 (-4.5, -1.1) | 0.001 | -3.5 (-4.9, -2.0) | <0.001 |  | -1.7 (-3.0, -0.3) | 0.02 | -0.5 (-1.4, 0.4) | 0.3 |  |
| Model 3 | -2.8 (-4.5, -1.1) | 0.001 | -3.5 (-4.9, -2.0) | <0.001 |  | -1.6 (-2.9, -0.3) | 0.02 | -0.4 (-1.3, 0.5) | 0.4 |  |

Model 1: adjusted for age and sex. Model 2 also adjusted for body mass index, smoking history and socioeconomic position. Model 3: as for model 2 plus adjustment for hypertension, diabetes, stroke, angina, myocardial infarction.

**Supplementary table 6b** Mean percentage difference in adipokines at age 60-64 by accumulation of leisure-time physical activity (LTPA) across adulthood – after multiple imputation.

|  | **Leptin (ng/ml)** | | | |  | **Adiponectin (ug/ml)** | | | |
| --- | --- | --- | --- | --- | --- | --- | --- | --- | --- |
|  | *Men (n=999)* | *P* | *Women (n=1057)* | *p-value* |  | *Men (n=998)* | *P* | *Women (n=1056)* | *p-value* |
| *Adulthood LTPA score (0-8): per 1-unit increase* |  |  |  |  |  |  |  |  |  |
| Model 1 | -3.5 (-5.6, -1.4) | 0.001 | -8.2 (-10.3, -6.1) | <0.001 |  | -0.6 (-2.5, 1.3) | 0.6 | 3.7 (2.0, 5.4) | <0.001 |
| Model 2 | -3.3 (-5.0, -1.6) | <0.001 | -3.0 (-4.7, -1.3) | <0.001 |  | -0.9 (-2.9, 1.1) | 0.4 | 1.8 (0.03, 3.5) | 0.05 |
| Model 3 | -3.2 (-4.9, -1.5) | <0.001 | -3.0 (-4.7, -1.3) | 0.001 |  | -1.2 (-3.2, 0.7) | 0.2 | 1.7 (-0.03, 3.4) | 0.06 |

Model 1: adjusted for age. Model 2 also adjusted for body mass index, smoking history and socioeconomic position. Model 3: as for model 2 plus adjustment for hypertension, diabetes, stroke, angina, myocardial infarction.

**Supplementary table 6c** Mean percentage difference in biomarkers at age 60-64 by change in leisure-time physical activity (LTPA) between ages 36 and 60-64 – after multiple imputation.

|  | Always inactive | Became inactive | Became active | Always active | p (overall association) |
| --- | --- | --- | --- | --- | --- |
| *C-reactive protein (n=2065)* |  |  |  |  |  |
| Model 1 | 0.0 | -13.7 (-24.0, -3.4) | -19.7 (-36.4, -3.0) | -30.4 (-41.3, -19.5) | <0.001 |
| Model 2 | 0.0 | -7.3 (-17.1, 2.4) | -7.0 (-23.1, 9.1) | -17.2 (-27.9, -6.5) | 0.02 |
| Model 3 | 0.0 | -7.8 (-17.5, 1.9) | -7.1 (-23.1, 9.0) | -17.3 (-28.0, -6.6) | 0.02 |
|  |  |  |  |  |  |
| *Interleukin-6 (n=2052)* |  |  |  |  |  |
| Model 1 | 0.0 | -8.0 (-16.3, 0.3) | -19.2 (-32.7, -5.7) | -24.8 (-33.7, -15.9) | <0.001 |
| Model 2 | 0.0 | -3.4 (-11.5, -4.8) | -9.3 (-22.6, 4.0) | -15.1 (-24.1, -6.1) | 0.004 |
| Model 3 | 0.0 | -3.3 (-11.4, 4.8) | -9.1 (-22.4, 4.1) | -14.6 (-23.6, -5.6) | 0.006 |
|  |  |  |  |  |  |
| *Tissue plasminogen activator (n=1798*) |  |  |  |  |  |
| Model 1 | 0.0 | 2.0 (-6.3, 10.4) | -4.5 (-17.0, 8.0) | -10.0 (-18.3, -1.7) | 0.01 |
| Model 2 | 0.0 | 3.9 (-4.3, 12.0) | -0.7 (-13.1, 11.7) | -5.5 (-13.8, 2.7) | 0.1 |
| Model 3 | 0.0 | 3.7 (-4.4, 11.8) | -0.6 (-13.0, 11.7) | -5.4 (-13.6, 2.8) | 0.1 |
|  |  |  |  |  |  |
| *E-selectin (n=2052)* |  |  |  |  |  |
| Model 1 | 0.0 | -0.5 (-5.7, 4.6) | -1.7 (-10.4, 7.0) | -4.5 (-9.9, 0.8) | 0.4 |
| Model 2 | 0.0 | 1.2 (-3.8, 6.3) | 1.4 (-7.1, 10.0) | -1.0 (-6.5, 4.4) | 0.8 |
| Model 3 | 0.0 | 1.3 (-3.7, 6.4) | 1.4 (-7.2, 9.9) | -0.8 (-6.2, 4.7) | 0.8 |
|  |  |  |  |  |  |
| Leptin – men (n=999) |  |  |  |  |  |
| Model 1 | 0.0 | 8.9 (-3.5, 21.3) | -0.4 (-24.0, 23.2) | -12.5 (-25.9, 0.9) | 0.004 |
| Model 2 | 0.0 | 1.4 (-8.0, 10.7) | -2.6 (-21.5, 16.3) | -15.2 (-25.6, -4.8) | 0.003 |
| Model 3 | 0.0 | 1.3 (-8.1, 10.6) | -1.6 (-20.5, 17.3) | -14.1 (-24.5, -3.6) | 0.007 |
|  |  |  |  |  |  |
| Leptin – women (n=1057) |  |  |  |  |  |
| Model 1 | 0.0 | -17.8 (-31.0, -4.7) | -29.3 (-48.5, -10.0) | -46.2 (-60.1, -32.4) | <0.001 |
| Model 2 | 0.0 | 0.1 (-9.5, 9.8) | -4.0 (-18.2, 10.1) | -16.0 (-26.6, -5.4) | 0.004 |
| Model 3 | 0.0 | 0.2 (-9.5, 9.9) | -4.1 (-18.2, 10.1) | -15.7 (-26.4, -5.1) | 0.006 |
|  |  |  |  |  |  |
| Adiponectin – men (n=998) |  |  |  |  |  |
| Model 1 | 0.0 | 1.0 (-10.6, 12.7) | -7.8 (-30.9, 15.3) | -6.4 (-18.9, 6.0) | 0.5 |
| Model 2 | 0.0 | 2.9 (-8.5, 14.3) | -7.6 (-30.5, 15.2) | -7.1 (-20.0, 5.3) | 0.3 |
| Model 3 | 0.0 | 2.4 (-8.9, 13.8) | -8.6 (-31.5, 14.2) | -9.0 (-21.4, 3.4) | 0.2 |
|  |  |  |  |  |  |
| Adiponectin – women (n=1056) |  |  |  |  |  |
| Model 1 | 0.0 | 9.5 (-0.4, 19.4) | 13.2 (-1.4, 27.6) | 22.0 (11.4, 32.6) | <0.001 |
| Model 2 | 0.0 | 2.6 (-6.9, 12.1) | 3.5 (-10.5, 17.5) | 10.8 (0.2, 21.4) | 0.2 |
| Model 3 | 0.0 | 1.9 (-7.6, 11.4) | 3.9 (-10.0, 17.8) | 10.5 (-0.1, 21.1) | 0.2 |

Model 1: adjusted for age (and sex). Model 2 also adjusted for body mass index, smoking history and socioeconomic position. Model 3: as for model 2 plus adjustment for hypertension, diabetes, stroke, angina, myocardial infarction.
